# Novel variants of *TP63* identified in Chinese families with split-hand/foot malformation

**DOI:** 10.3389/fgene.2026.1855600

**Published:** 2026-07-16

**Authors:** Xuyu Gu, Siyuan Tao, Xiaodong Wang, Xiuli Zhao, Xiaofang Shen

**Affiliations:** 1 Key Laboratory in Science and Technology Development Project of Suzhou (CN), Pediatric Orthopedics, Children’s Hospital of Soochow University, Suzhou, China; 2 Center for Rare Diseases, State Key Laboratory of Complex, Severe, and Rare Diseases, Peking Union Medical College Hospital, Chinese Academy of Medical Sciences & Peking Union Medical College, Beijing, China; 3 Department of Medical Genetics, State Key Laboratory for Complex, Severe, and Rare Diseases, Institute of Basic Medical Sciences, Chinese Academy of Medical Sciences, School of Basic Medicine, Peking Union Medical College, Beijing, China

**Keywords:** DNA sequencing, genetic diagnosis, split-hand/foot malformation, TP63, variant

## Abstract

**Objective:**

Split-hand/foot malformation (SHFM) is a group of congenital birth defects affecting the hands and feet, significantly impairing patients quality of life. The *TP63* gene encodes the p63 protein, heterozygous *TP63* variants can cause SHFM. The aim of this study was to identify *TP63* gene variants in three Chinese families with SHFM.

**Methods:**

Three Chinese families with SHFM enrolled in this study. Proband 1 and Proband two had familial history of SHFM, whereas Proband 3 was a sporadic case of this disease. Peripheral blood was collected from the proband and their available family members for genomic DNA extraction. Candidate pathogenic variants were identified through whole exome sequencing (WES), validated using PCR-based Sanger sequencing and bioinformatic analysis.

**Results:**

We found three different missense variants in the *TP63* gene: c.2032G>C (p.Glu678Gln), c.956G>A (p.Arg319His), and c.689T>A (p.Val230Asp). Among them, c.689T>A (p.Val230Asp) is novel variant.

**Conclusion:**

In the present work, three *TP63* gene variants were found in the families with SHFM, which expanded the variant spectrum of *TP63*. These findings not only provide further evidence for their heterogeneous role in limb and non-limb malformations but also broaden the genetic spectrum of associated disorders. These insights pave the way for improved prenatal genetic diagnosis and informed counseling.

## Introduction

Split-hand/foot malformation (SHFM) is a severe congenital limb disorder, primarily characterized by the absence or underdevelopment of the central rays of the hands and/or feet. Affecting families worldwide, the incidence of SHFM is approximately 0.51–0.60 per 10,000 live births, as reported in epidemiological studies (Duijf et al., 2003; Elliott et al., 2005). The pronounced clinical heterogeneity of SHFM has led to the association of multiple genes and subtypes with the non-syndromic form, including *DLX5* (SHFM1), Xq26 (SHFM2), dup10q24 (SHFM3), *TP63* (SHFM4), del2q31 (SHFM5), *WNT10B* (SHFM6), and dup17p13.3 (SHFM/SHFLD) (Sowinska-Seidler et al., 2014; Umair and Hayat, 2020).

Limb development proceeds along the proximodistal axis through sequential, layered differentiation. This process depends on three main structures: the Apical Ectodermal Ridge (AER), Progress Zone (PZ), and Zone of Polarizing Activity (ZPA) (Merlo et al., 2002; Sidow et al., 1997). The AER secretes fibroblast growth factors (FGFs) to maintain the proliferation and undifferentiated state of nearby mesenchymal cells, enabling limb outgrowth (Yang et al., 1999). Disruption of these signals leads to limb malformations, as demonstrated by AER removal studies (Duijf et al., 2003).

*TP63* gene encodes the p63 protein, which is involved in transcriptional activation, cell cycle arrest, and apoptosis (Rinne et al., 2007; Yang and McKeon, 2000). This gene is essential for maintaining AER integrity during embryonic development (Amiel et al., 2001; McDade et al., 2012; Candi et al., 2006; Bakkers et al., 2002). In mouse models, *TP63* knockout reduces cell proliferation in the central AER (Crackower et al., 1998)and disrupts normal cellular growth and differentiation (van Bokhoven et al., 2002; Dohn et al., 2001), resulting in deficits that ultimately prevent proper development of the central limb rays. This defect constitutes the core pathogenic mechanism of SHFM4 due to *TP63* variants.

In this study, we investigated three different *TP63* gene variants identified in three Chinese families with SHFM4. Candidate variants were identified by WES and verified by Sanger sequencing. We totally identified ten families with pathogenic variants of *TP63*, including two new variants. Broadens the known spectrum of *TP63* alterations. These findings enhance our understanding of the genetic basis of SHFM and underscore the importance of *TP63* screening in the molecular diagnosis of this disorder.

## Materials and methods

### Clinical examination

This study enrolled over 50 pedigrees with congenital limb malformations from outpatient and inpatient clinics between 2024 and 2025. The study protocol was approved by the local ethics committee, and informed consent was obtained from all participating family members. Diagnoses were based on radiographic (X-ray), computed tomography (CT), and magnetic resonance imaging (MRI) findings, combined with clinical manifestations, following the ICD-10 criteria.

### Subjects and DNA samples

There are over 50 families with congenital limb malformations which were collected from outpatient clinics between 2023 and 2025 in this study. All of the probands and their families underwent clinical evaluations, including physical examinations and X-ray examinations, prior to surgery. The probands and all available family members’ peripheral blood samples (3–5 mL) were obtained. Genomic DNA was extracted using the conventional proteinase K - phenol - chloroform method. (Tao et al., 2025). Among them, three families were confirmed to have the variants of *TP63* gene and were included in this study.

### Whole exome sequencing (WES)

For all probands, WES were conducted according to the following workflow: exome capture was carried out with the Agilent Sure Select Human All Exon kit (Agilent Technologies, Wilmington, DE). Following ultrasonic fragmentation of DNA into segments of approximately 150–300 bp, a capture DNA library was prepared and sequenced by the HiSeq 2000 platform (Illumina, Inc. from San Diego, California). Raw data collection, including error assessment and base calling, was performed using Illumina Pipeline (version 1.3.4). To identify sequence variants in disease-associated genes, targeted next-generation sequencing was employed. The obtained sequence reads were aligned against the human reference genome (hg19) from the UCSC database. Variants were filtered by referencing the Single Nucleotide Polymorphism Database (dbSNPs) and the 1000 Genomes Project database. Reported pathogenic variants were subsequently confirmed using entries from the Human Gene Mutation Database (HGMD, http://www.hgmd.cf.ac.uk/ac/index.php) maintained by the Institute of Medical Genetics in Cardiff, or based on previously published literature. Finally, the clinical relevance of variants was evaluated following established criteria such as the ACMG guidelines to assess their potential to explain the patient’s phenotype.

### PCR-sanger sequencing

To validate candidate variants identified through WES and to assess genotype-phenotype co-segregation within families, PCR-Sanger sequencing was employed. Reference sequences for *TP63* genomic DNA (cDNA) and protein (hg19: NM_003722.5, NP_003713.3) were acquired from the UCSC Genome Browser (http://genome.ucsc.edu/) and the NCBI Reference Sequence Project. Primers were designed using the online tool Primer3 (http://primer3.ut.ee/) and subsequently verified via the UCSC Genome Browser BLAT and *in silico* PCR tools (Table 1). All PCR reactions were performed with TaKaRa LA Taq® with GC Buffer (TaKaRa, Shiga, Japan) under the following conditions: initial denaturation at 95 °C for 3 min; 35 cycles of 94 °C for 30s, 58 °C for 40s, and 72 °C for 40s; and a final extension at 72 °C for 8 min. Sequencing was conducted on an Applied Biosystems 3730xl DNA Analyzer (Thermo Fisher Scientific, Waltham, MA, United States of America), and the resulting data were aligned to the reference sequence using Codon Code Aligner (v.6.0.2.6; Codon Code, Centerville, MA, United States of America) (Table1).

**TABLE 1:** The primers for PCR and sanger sequencing in pathogenic variant validation.

Proband	Mutation	Forward primer (5′-3′)	Reverse primer (5′-3′)	Product length (bp)
1	c.2032G>C	TCC​ACG​AAT​TCT​CCT​CCC​CT	TGC​AGC​TTA​AGG​AGA​CAC​CC	501 bp
2	c.956G>A	AACAGGGTTCAGAGTTTGCC	CCACCTTCCTTTGTCTCCAC	434 bp
3	c.689T>A	TTTAAACCCCTCTGAGCCTCA	AGAAGGAAGCAGGAAGGAGT	763 bp

### Microsatellite marker analysis

To confirm the biological relationship between the proband and his parents in Family 3, three highly polymorphic microsatellite markers (D17S579, D17S156582, and D2S1391). PCR amplification was performed using fluorescently labeled forward primers (Supplementary Table S1). The PCR products were subjected to capillary electrophoresis on an ABI 3730xl DNA Analyzer, and allele sizes were determined using GeneMapper v5.0 software. The results confirmed paternity and maternity with a probability >99.9%.

### Pathogenic analysis

Candidate variants were assessed for potential functional impact following the ACMG guidelines. The VarCards2 platform (https://genemed.tech/varcards2), which integrates over 30 computational algorithms for allele frequency filtering, pathogenicity prediction, and database annotation, was utilized for this analysis. For identified missense variants, evolutionary conservation across species was analyzed using MEGA-11 (Molecular Evolutionary Genetics Analysis version 11). Subsequently, the three-dimensional structures of both wild-type and mutant TP63 proteins were modeled with Swiss-Model and visualized using Swiss-PdbViewer software. The structural models were generated using the following PDB templates: for the DNA-binding domain (residues 153–384), PDB:2RMN (solution structure, 1.80 Å) and PDB:7Z7E (crystal structure, 1.80 Å) were used as references; for the C-terminal SAM domain (residues 540–614), PDB:1RG6 (solution structure) was used as the template.

## Results

### Clinical analysis

**FIGURE 1:**
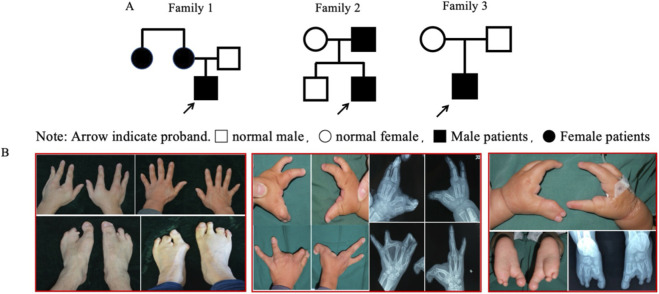
The pedigrees and clinical manifestations of the three probands. **(A)** Pedigrees of the three families. **(B)** Clinical manifestations in the probands and some affected parents.

Three families with limb malformations were caused by *TP63* variants. The pedigrees and clinical manifestations were presented in Figure 1 and Table 1. Proband 1 had bilateral absence of the middle fingers and third metacarpals, and also expressed the missed third toes and partial third metatarsals. The proband’s mother and aunt showed the absence of the phalanges in both feet. Proband two showed malalignment of the index and middle fingers. The third toe was corresponding metacarpal involvement on both hands. His father had a similar phenotype but with more complex metacarpal malalignment. Proband 3 demonstrated bilateral SHFM (split hand/foot malformation) featuring central ray deficiency (absence of middle digits) and absence of bone elements. No similar phenotype was observed among other family members.

### Genetic identification

**FIGURE 2:**
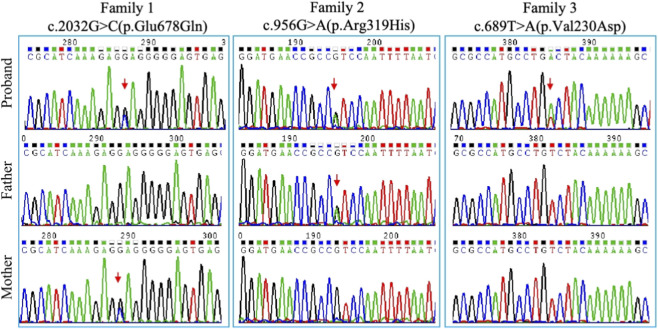
*TP63* variants identified in the three families with SHFM. Sanger sequencing results of the three families.

In Family 1, the heterozygous variant c.2032G>C (p.Glu678Gln) in exon 14 of *TP63* was found in the proband, his mother, and aunt. In Family 2, the variant c.956G>A (p.Arg319His) in exon 7 of *TP63* was present in both the proband and his father. In Family 3, the proband 3 was heterozygous for the c.689T>A (p.Val230Asp) variant in exon 5 of *TP63*. Neither parent carried this alteration (Figure 2). Microsatellite marker analysis confirmed the biological relationship between the proband and both parents (probability >99.9%), supporting a *de novo* origin of this variant.

### Bioinformatic analysis

**FIGURE 3:**
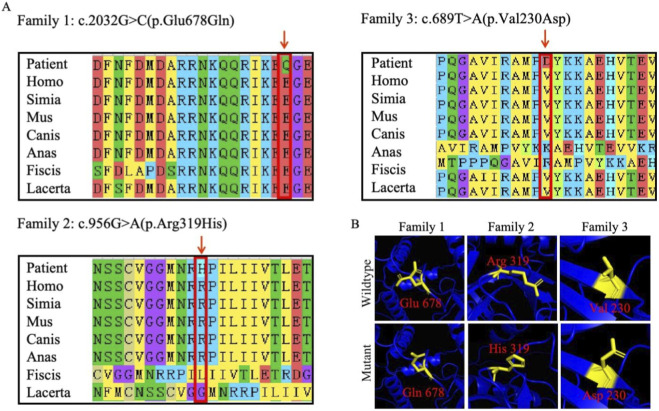
Bioinformatics analysis of three novel missense variants of TP63. **(A)** Evolutionary conservation of amino acids at variant sites. The variant sites are marked with red boxes. **(B)** Three-dimensional structural comparisons between mutant and wild types.

Evolutionary conservation analysis using MEGA12 was performed to compare TP63 amino acid sequences across multiple mammalian species from the NCBI database. The region surrounding each variant site—spanning 20 amino acids, showed >95% identity across diverse species including mammals, birds, and some reptiles (Figure 3A). With the exception of fish, overall sequence identity reached nearly 98%, supporting high evolutionary conservation (preservation across species) of TP63, as previously reported (van Bokhoven et al., 2002), and suggesting strong pathogenic (disease-causing) potential for variants in this gene (Table 2). Pathogenicity prediction (evaluation of whether a genetic change causes the disease) via Variant Taster classified all three variants as “disease-causing”. Potential mechanisms are illustrated in Table 2, reinforcing their association with SHFM. Protein structural modeling was performed using SWISS-MODEL based on PDB templates 2RMN (DNA-binding domain, 1.80 Å) and 1RG6 (C-terminal SAM domain). For the p. Val230Asp variant in the DNA-binding domain, the substitution of the hydrophobic valine residue (Val230) by a negatively charged aspartate is predicted to disrupt local hydrophobic packing within the DBD core. Val230 is located in a structurally conserved region adjacent to the DNA-binding interface; its replacement by a charged residue may alter side-chain orientation and affect the local backbone conformation, potentially impairing DNA-binding capacity. For the p. Glu678Gln variant in the C-terminal transactivation inhibitory domain, the substitution of a negatively charged glutamate by a neutral glutamine eliminates a potential ubiquitination site. Structural modeling indicates that this change is restricted to local side-chain alterations without affecting the overall fold of the SAM domain or the TID region. These predictions, together with ubiquitination site analysis (see Discussion), support the deleterious effects of these variants. (Figure 3B).

**TABLE 2:** Clinical symptoms and information on the variants in the three *TP63* variants families.

Proband	Age	Clinical symptoms	Variant type	Nucleotide change	Amino acid change	ACMG criteria	Pathogenicity	Novel or reported
1	35 years	Absence of the middle finger and the third metacarpal; loss of the third toe, and partial loss of the third metatarsal	Missense	c.2032G>C	p.Glu678Gln	PS1+PM1+PM2_Supporting+PP1_Moderate+PP3	Pathogenic	Reported
2	1 year	The index and middle fingers are misaligned, and both hands’ metacarpals are affected	Missense	c.956G>A	p.Arg319His	PS1+PM1+PM2_Supporting+PP1_Moderate+PP3	Pathogenic	Reported
3	1 year	Central ray missing; absence of the middle finger	Missense	c.689T>A	p.Val230Asp	PM2_Supporting +PS2+PP3	Likely pathogenic	Novel

Pathogenicity evaluation of candidate variants according to ACMG guidelines.

The evidence codes applied and the final pathogenicity classification for each variant are summarized in Table 2.

All three missense variants were evaluated according to the ACMG 2015 guidelines. For missense variants, the pathogenic classification typically requires the combination of multiple evidence types; PM2 alone is applied at the supporting level (PM2_Supporting). The c.2032G>C (p.Glu678Gln) variant, which has been previously reported, was classified as Pathogenic (Yang et al., 2025). The c.956G>A (p.Arg319His) variant, which has been previously reported, was classified as Pathogenic (refer to existing literature) (van Bokhoven et al., 2001). The c.689T>A (p.Val230Asp) variant was classified as Likely Pathogenic based on PM2_Supporting, PS2 (Supplementary Figure S1), and PP3.

## Discussion

SHFM is genetically heterogeneous, categorized into six types with distinct inheritance patterns: autosomal dominant (SHFM1, 3, 4, 5, SHFLD), X-linked recessive (SHFM2), and autosomal recessive (SHFM6) (Sowinska-Seidler et al., 2014; Wright et al., 2019). This heterogeneity is further reflected in the clinical spectrum, where non-limb phenotypes are common in SHFM4/5, and long bone dysplasia in SHFM6/SHFLD (Umair and Hayat, 2020; Elliott and Evans, 2006). Consequently, accurate subtyping directs a more focused diagnostic approach and provides a foundation for tailored patient management.

According to the HGMD Professional 2024.4 database, a total of 187 *TP63* variants have been reported across all associated disorders. Among these, 25 distinct mutations have been associated with SHFM4, including 18 missense/nonsense mutations, two splicing mutations, 3 gross deletions, and two small deletions/insertions (HGMD Professional 2024.4). The distribution of SHFM4-associated mutations across the *TP63* gene is more widespread compared to other p63-related disorders, consistent with previous observations. SHFM is a heterogeneous limb malformation that can occur alone or with other congenital anomalies (Umair and Hayat, 2020; Elliott and Evans, 2006). In this study, all three families presented with *TP63*-related SHFM4, with affected individuals displaying classic central ray deficiencies and bone hyperplasia. Notably, the abnormalities were restricted to ectodermal-derived structures, and no intellectual impairment or cardiac involvement was observed—consistent with known *TP63*-related phenotypic spectra (Elliott and Evans, 2006).

In Family 1, the proband carried a c.2032G>C (p.Glu678Gln) variant, which is predicted to alter protein function. A previously reported change at the same site, c.2032G>T (p.Glu678Ter), also lead to SHFM4 (van Bokhoven et al., 2002), supporting the pathogenic effect of this residue. This finding aligns with the phenotype observed in this study. The proband from Family 3 carried a c.689T>A (p.Val230Asp) variant, which may impact local protein structure. Neighboring variants such as c.670G>A (p.Val224Ile) (Khandelwal et al., 2019) and c.691T>G (p.Tyr231Asp) (Rinne et al., 2007) have been linked to cleft lip/palate, indicating sensitivity of this region. Although no overt orofacial cleft was observed in this proband, mild or subclinical manifestations cannot be ruled out. The role of variants in this region in cleft pathogenesis warrants further investigation.

Family two carries a variant that has been reported before. Previous studies have found that this variant can cause symptoms like sparse hair, problems with the tear ducts, dry skin, and missing teeth (van Bokhoven et al., 2001). Although our patient did not show these features, the variant might still influence how the condition presents itself in different parts of the body. More research with larger groups of patients and laboratory experiments is needed to better understand how this variant leads to different symptoms. The main limitation of this study is that the genetic status of the patients was initially unknown, requiring diagnosis based solely on clinical presentation.

It is noteworthy that the novel variant p. Glu678Gln resides in the C-terminal region of *TP63*, specifically within the transactivation inhibitory domain (TID). Previous studies have shown that the C-terminus of p63 contains lysine residues critical for ubiquitin-mediated proteasomal degradation, and mutations in this region primarily affect protein stability rather than global structure (van Bokhoven et al., 2001). Notably, the two novel variants are located in different functional domains of p63. The p. Glu678Gln variant lies in the C-terminal transactivation inhibitory domain (TID), within a region (residues 656–680) recently identified as a SHFM4-specific hotspot. A known mutation at the same residue (p.Glu678Ter) has been reported to cause SHFM4, supporting our finding. The p. Val230Asp variant lies in the DNA-binding domain (DBD). While most DBD mutations cause syndromic EEC syndrome, a subset of DBD mutations can cause isolated SHFM4, which is consistent with the phenotype observed in Family 3. Mechanistically, C-terminal SHFM4 mutations maintain near-normal protein stability and transcriptional activity, whereas DBD mutations typically reduce transcriptional activity. This dichotomy explains why C-terminal mutations often cause isolated limb defects, while DBD mutations tend to cause syndromic forms.

Using *in silico* tools (UbPred, NetSurfP-3.0), we predict that Glu678 lies within a potential ubiquitination site; substitution to Gln may alter ubiquitin conjugation efficiency, thereby impacting p63 turnover. Determining whether p. Glu678Gln causes gain or loss of function will require experimental validation (e.g., protein stability assays). Nevertheless, the location of this variant is consistent with the mild limb-restricted phenotype observed in Family 1, as C-terminal *TP63* mutations are often associated with isolated SHFM rather than syndromic forms.

### Study limitations

Several limitations of this study should be acknowledged. First, the sample size is small, with only three families included. Given the rarity of split-hand/foot malformation (SHFM), larger cohort studies are needed to fully characterize the phenotypic spectrum and genotype–phenotype correlations associated with these *TP63* variants. Second, functional experiments are lacking. We did not perform *in vitro* or *in vivo* functional validation (e.g., transcriptional activity assays, protein stability analyses, or oligomerization studies) for the novel missense variants. Therefore, the precise molecular mechanisms by which these variants disrupt p63 function remain to be elucidated. Future studies will employ luciferase reporter assays to assess the transactivation activity of these *TP63* variants on p63-responsive promoters, as well as protein stability assays to evaluate whether these missense changes affect p63 half-life or subcellular localization. Such functional validation will be essential to fully establish pathogenicity and to clarify the genotype–phenotype correlations of *TP63* related disorders.

## Conclusion

This clinical and genetic study of three pedigrees revealed significant clinical heterogeneity among affected individuals and identified one novel pathogenic variants. Patients have clinical heterogeneity. These findings provide a genetic basis for precise diagnosis and genetic counseling for the related families with SHFM, although functional validation of the novel variants is needed to fully establish pathogenicity.

## Data Availability

The original contributions presented in the study are publicly available. This data can be found in the ClinVar repository with the accession numbers VCV003233406.3, VCV000265276.8, and VCV004755565.1.
