# Supplementary material for: Novel variants of TP63 identified in Chinese families with split-hand/foot malformation
Source: Front Genet. 2026 Jul 16;17:1855600. doi: 10.3389/fgene.2026.1855600 (PMC13427037; doi:10.3389/fgene.2026.1855600)
Supplement: Supplementary file 1 [file Table1.pdf]

Table S1. Microsatellite markers used for paternity confirmation in Family 3

STS Marker	Primer	
D17S579	F	5’-AGTCCTGTAGACAAAACCTG-3’
	R	5’-CAGTTTCATACCAAGTTCCT-3’
D17S156582	F	5’-CAGAGTCTGGCACATACATGAA-3’
	R	5’-TCCTCTCAACATGACATTCCCT-3’
D2S1391	F	5’-CTCACTGTCTGGATTTCTTGG-3’
	R	5’-TAAGGACAAGTTAAAAAAGCTGG-3’
